# The Impact of the February 2012 Cold Spell on Health in Italy Using Surveillance Data

**DOI:** 10.1371/journal.pone.0061720

**Published:** 2013-04-18

**Authors:** Francesca K. de'Donato, Michela Leone, Damia Noce, Marina Davoli, Paola Michelozzi

**Affiliations:** 1 Department of Epidemiology, Lazio Regional Health Service, Rome, Italy; 2 National Centre for the Prevention of Heat Health Effects, Department of Civil Protection, Rome, Italy; Public Health Agency of Barcelona, Spain

## Abstract

In February 2012 Italy was hit by an exceptional cold spell with extremely low temperatures and heavy snowfall. The aim of this work is to estimate the impact of the cold spell on health in the Italian cities using data from the rapid surveillance systems. In Italy, a national mortality surveillance system has been operational since 2004 in 34 cities for the rapid monitoring of daily mortality. Data from this system were used to evaluate the impact of the February 2012 cold spell on mortality shortly after the occurrence of the event. Furthermore, a cause-specific analysis was conducted in Roma using the Regional Mortality Registry and the emergency visits (ER) surveillance system. Cold spell episodes were defined as days when mean temperatures were below the 10^th^ percentile of February distribution for more than three days. To estimate the impact of the cold spell, excess mortality was calculated as the difference between observed and daily expected values. An overall 1578 (+25%) excess deaths among the 75+ age group was recorded in the 14 cities that registered a cold spell in February 2012. A statistically significant excess in mortality was observed in several cities ranging from +22% in Bologna to +58% in Torino. Cause-specific analysis conducted in Roma showed a statistically significant excess in mortality among the 75+ age group for respiratory disease (+64%), COPD (+57%), cardiovascular disease +20% ischemic heart disease (14%) and other heart disease (+33%). Similar results were observed for ER visits. Surveillance systems need to become are a key component of prevention plans as they can help improve public health response and are a valid data source to rapidly quantify the impact on health. Cold-related mortality is still an important issue and should not be underestimated by public health Authorities.

## Introduction

Between January and February 2012 cold polar air from Siberia brought extremely low temperatures and heavy snowfall across most of Europe [1]. Italy suffered an exceptional cold spell; both in terms of the low temperatures and persistence of these conditions that had not been registered since 1985. Record low temperatures, were registered in north-eastern Italy while snow depths of up to 3 meters were recorded in central regions. Entire communities were isolated and cut off from energy and food supplies for several days, principally due to the snow. The media reported deadly events associated to the cold spell, in particular for hypothermia but an overall assessment of the impact of the cold spell is still missing.

Near-real time surveillance systems are useful to monitor trends in health outcomes and detect anomalous peaks. Systems have been developed to monitor mortality [2], non-fatal outcomes [3]–[6] as well as specific syndromic events, like influenza [7]. More recently, surveillance systems have also been used to specifically monitor the health effects of environmental exposures such as heat waves [3], [6], [8], [9] and to give rapid estimates of the health effects of extreme events. In Italy, a rapid mortality surveillance system was set up in 2004 in collaboration with Municipal Registry Offices to evaluate the impact of heat waves on health during summer [9]. This system was gradually extended to cover the whole year and to date has permitted the routine evaluation of heat waves throughout the summer as well as the timely evaluation of other extreme events like the cold spell of February 2012. An emergency room (ER) surveillance system was also set up to monitor non-fatal health effects of heat waves among the elderly in 15 cities in 2012 [10]. In the literature there are several case studies of the use of ER visits for monitoring and evaluating the effects of heat waves on non-fatal health outcomes [8], [11]–[13]. However, there is limited evidence of the use of ER visit data to monitor the effect of low temperatures and cold spells in winter. The surveillance of morbidity indicators are of great importance in public health to plan prevention strategies and define preparedness protocols to cope with emergencies.

Several studies have shown the effect of low temperatures and cold spells on health with peaks in both mortality, hospital admissions and ER visits [11], [14]–[19], in particular for respiratory and cardiovascular disease [14], [20], [21]. In the winter season, mortality fluctuations have been attributed to weather, seasonal patterns of illnesses, especially for influenza and socio-demographic characteristics of local populations [20], [22], [23]. The lag between cold weather and its impact on health is usually longer than that observed for heat and shows a greater variability by cause. Cardiac events peak within the first few days, while respiratory disease have a longer lag of up to 2 weeks [14]–[17], [24]. Although epidemiological studies in Europe have shown that the greatest impact of cold is in temperate regions where populations are unprepared to cope with these extreme conditions [14]–[15] in Italy, there is no cold prevention plan in place due to the limited occurrence of cold spells.

The aim of this work was to provide a rapid estimate of the impact of the February 2012 cold spell on mortality in Italian cities using data from surveillance systems. For Rome only, mortality and ER visits by cause were analysed to better characterise the differential impact of cold on subjects with specific illnesses.

## Methods

### Exposure measure

Meteorological data refer to the airport stations located closest to the city centre and were obtained from the Meteorological Service of the Italian Air Force. Mean temperature was considered as the exposure variable and cold spell episodes were defined as at least three consecutive days with mean temperatures below the 10^th^ percentile of the February monthly distribution of a reference period (1995–2010).

### Outcome measure

The Italian mortality surveillance system was developed for the “near real-time” monitoring of daily mortality in all cities with more than 200,000 inhabitants and to date includes 33 municipalities [9]. Every day, mortality data for the resident population is sent by local Municipal Registry Offices to the National Coordination Centre (Department of Epidemiology, Regional Health Authority Roma). Individual records include: date of birth and death, gender, place of death, municipality of birth, residence and death, and the cause of the event (accidental\non-accidental). The dataset can be considered complete, on average, 72 hours after the day of death is recorded. Data from this system for 27 cities for which environmental data was also available, were used to evaluate the impact of the February 2012 cold spell on mortality.

In order to investigate the potential biological mechanisms associated with the exposure to cold, cause-specific mortality data and ER visits, for Roma only were considered. Mortality data was retrieved from the Lazio Regional Mortality Registry, while ER visits were retrieved from the ER surveillance system. In 2012, a national ER surveillance system was set up to evaluate the potential impact of heat on non-fatal health outcomes during summer. ER visits by cause were examined for Roma only as this data was already available from the ER surveillance system during the February 2012 cold spell.

Causes taken into account were: all natural causes (ICD-IX: 1-799), cardiovascular disease (ICD-IX: 390-459), ischemic heart disease (ICD-IX: 410-414), acute myocardial infarction (ICD-IX: 410-412), other heart disease (ICD-IX: 420-429), cardiac dysrhythmias (ICD-IX: 427), heart failure (ICD-IX: 428) respiratory disease (ICD-IX: 460-519), chronic obstructive pulmonary disease (ICD-IX: 490-496), acute respiratory infections (ICD-IX: 464-466 and 480-487) and injuries (ICD-IX: 800-959).

### Statistical analysis

As preliminary exploratory analysis, the mortality and ER visit trends were inspected to see whether the mortality during February 2012 was an exceptional event. A smoothing sine wave component was fitted to the dataset to account for seasonality trends in mortality, reducing noise associated with daily mortality variations in the data. For the identification of significant peaks in mortality, an upper limit was constructed using the Farrington method [25]. This method takes into account the asymmetric distribution of the data and the Poisson distribution over-dispersion and was defined using the methodology developed by Cox et al 2010 [2]. The upper limit was set to 100(1-α)% prediction interval, where α was set to 0.005.

Secondly, to estimate the health impact of the cold spell on mortality, the excess was calculated as the difference between observed daily deaths and daily expected values from the beginning of the cold spell to the end of the month to account for a possible delay in the effect between exposure to cold and effect typically observed during winter. This approximates the lag period generally considered in time series studies of the effect of low temperatures on mortality [14], [17], [26]–[28]. Expected daily mortality was defined as the mean daily value by calendar week and day of the week of the historical time series available (2008–2011). Total excess mortality was also calculated for the entire winter season (december 2011–March 2012) to account for the overall winter mortality during the period in study.

For the cause-specific analysis conducted in Roma, excess mortality and excess ER visits were defined as the difference between observed and expected counts by cause for the month of February. As for mortality, the excess in ER visits was defined as difference between daily observed and expected values. The latter was constructed as mean value by calendar week and day of the week during the reference period of four previous winter seasons (2008–2011).

## Results

**Figure 1:**
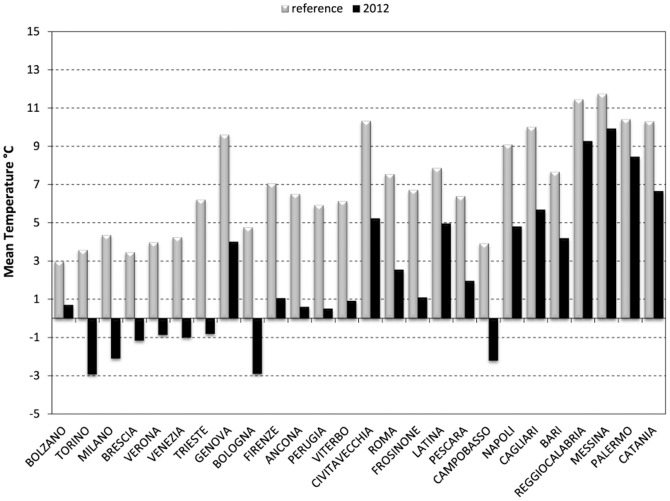
Mean Temperatures during the month of February 2012 and reference period.

Figure 1 shows mean temperatures during February of 2012 and in the reference period (1995–2010). Mean daily temperatures were much lower than the reference period in most northern and central cities, while in the south the temperature anomalies were limited. The persistence of the cold spell ranged from 16–18 days in northern cities to 7–15 days in central cities, in the south only a few cities registered a cold spell which was generally or shorter duration. Temperatures during the cold spell were much colder in the north and central areas while in southern areas the differences were less discernible (table 1).

**Table 1:** Observed, excess and percent variation in mortality among the very old (75 years and over) during the February 2012 cold spell episode in Italian cities.

City	Cold spell days	Temperature anomaly*	Observed	Excess	% variation	P-value
**Bolzano**	8	−4.7	88	28	47	**0.003**
**Torino**	17	−7.2	657	241	58	**<0.001**
**Milano**	15	−7.7	973	280	40	**<0.001**
**Brescia**	16	−5.5	160	41	34	**0.001**
**Verona**	16	−5.5	230	62	37	**<0.001**
**Venezia**	18	−5.5	308	65	27	**<0.001**
**Trieste**	17	−7.9	281	76	37	**<0.001**
**Genova**	17	−7.2	760	213	39	**<0.001**
**Bologna**	18	−7.6	371	66	22	**0.001**
**Firenze**	15	−6.9	323	32	11	0.075
**Ancona**	15	−6.7	82	17	26	0.06
**Perugia**	16	−6.1	118	29	33	**0.008**
**Roma**	14	−5.9	1829	406	29	**<0.001**
**Viterbo**	6	−6.3	21	−14	−40	0.002
**Civitavecchia**	7	−6.3	38	9	31	0.144
**Frosinone**	18	−5.3	38	9	31	0.144
**Latina**	13	−3.9	67	33	97	**<0.001**
**Pescara**	14	−5.3	86	22	34	**0.018**
**Campobasso**	14	−7.2	33	5	18	0.384
**Napoli**	11	−5.1	536	−30	−5	0.195
**Cagliari**	12	−5.5	1	−83	−99	<0.001
**Bari**	10	−4.8	150	14	10	0.253
**Reggiocalabria**	11	−3.5	92	−18	−16	0.061

* mean temperature (°C) difference during the cold spell days of 2012 and reference period.

**Figure 2:**
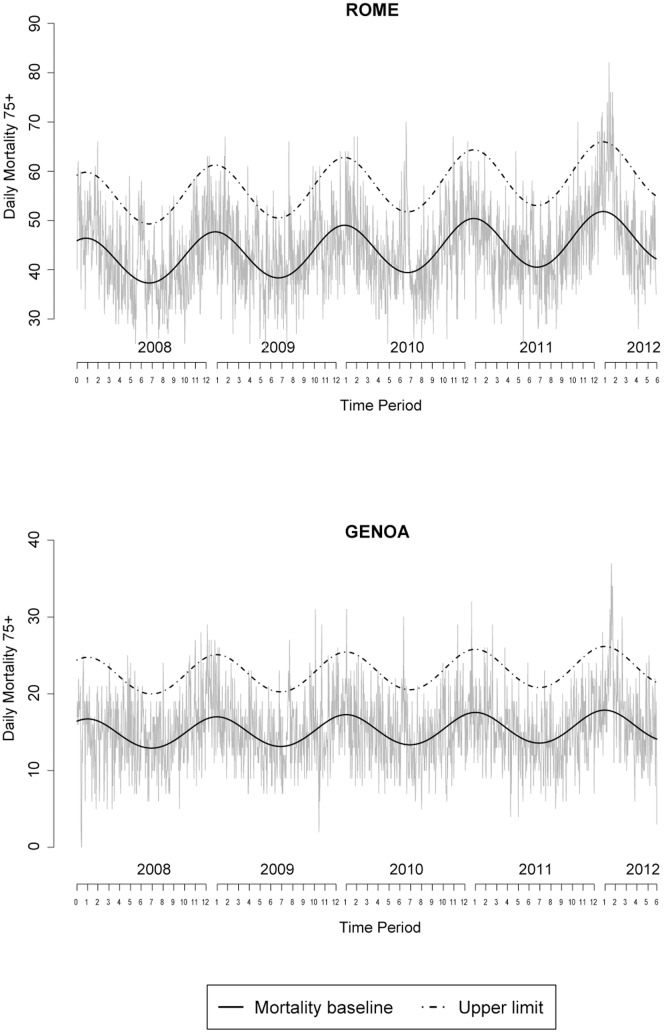
Daily Mortality trend (January 2008–June 2012) for the very old age group (75 years and over) in Roma and Genoa.

**Figure 3:**
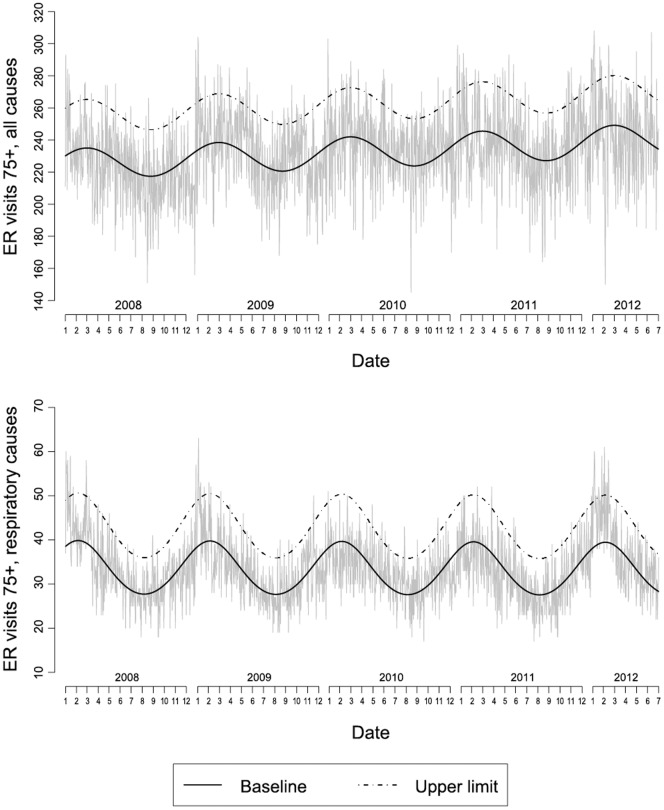
Daily trend (January 2008–June 2012) in ER visits for all causes and respiratory causes among the very old (75 years and over) in Roma.

Figure 2 shows the temporal trend in daily mortality in the 75+ age group between January 2008 and June 2012 in Roma and Genoa. A strong seasonal pattern can be observed with highest values in winter and troughs in summer. Peaks in mortality are those when mortality goes over the upper limit (dashed line), episodes worth mentioning are peaks in mortality during the 2010 heat wave, and the February 2012 cold spell episode for both cities. A similar trend was observed in other cities (data not shown). Figure 3 shows the temporal trend in ER visits for all causes (top) and respiratory causes (bottom) in the 75 and over age group for Roma between 2008 and June 2012. The seasonal trend in ER visits is similar to that observed for mortality, with peaks in winter and troughs during summer. However, total number of ER visits seem to less variable by season, while visits by respiratory causes are highly seasonal. A peak in February 2012, above the upper limit, was depicted for respiratory ER visits, while for total ER visits it was less evident and short-lived.

Table 1 shows the observed and excess mortality in the 75+ age group in all cities with a cold spell. A statistically significant excess in mortality was observed in several cities ranging from +22% in Bologna to +58% in Torino. In southern cities, where temperatures were milder, no significant excess in mortality was observed during the cold spell. Overall, results from the national surveillance system, showed an impact mainly in northern and central cities among the 75+ age group, with a total of 1578 (+25%) excess deaths recorded in the 15 cities with a cold spell during February 2012.

**Figure 4:**
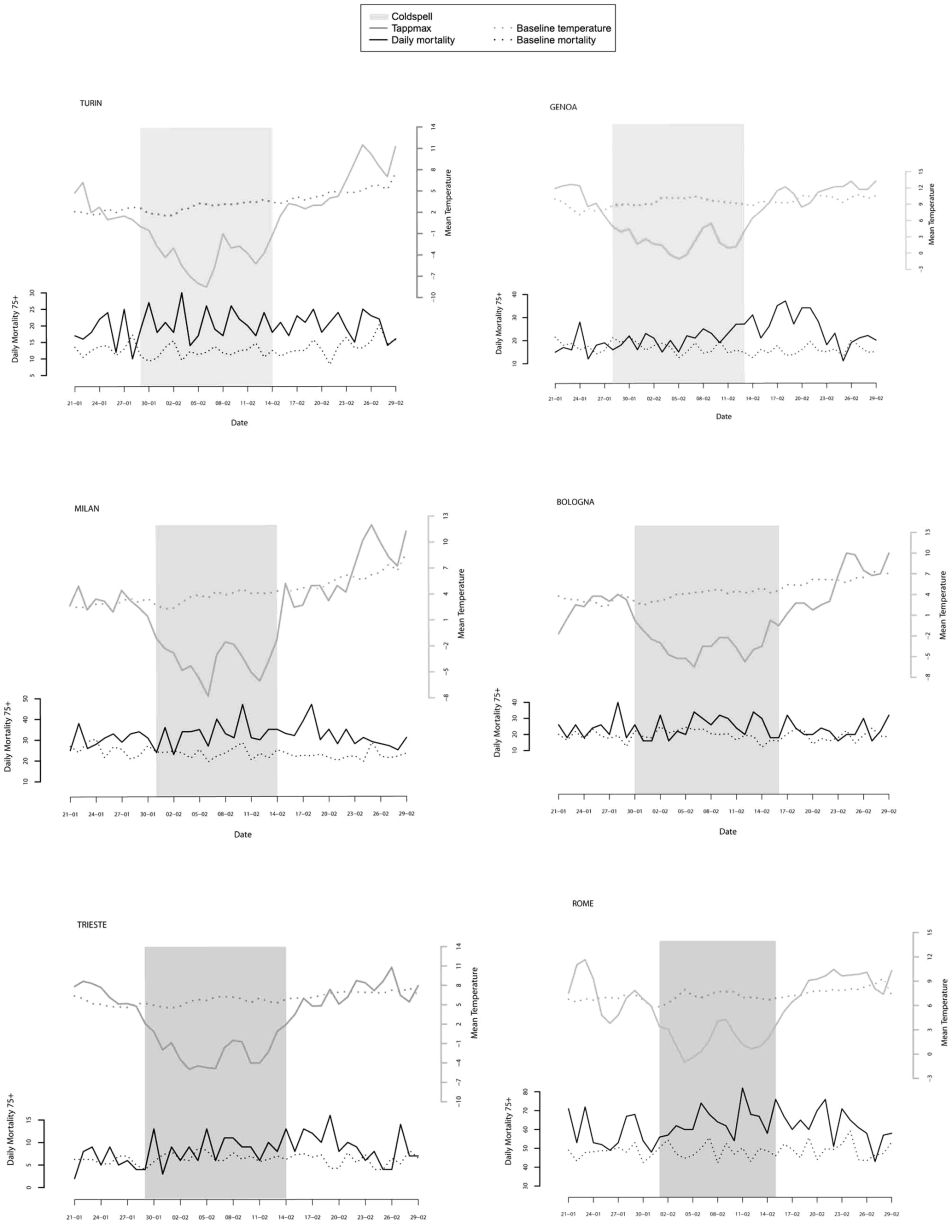
Daily mean temperature, observed and expected mortality during the cold spell of February 2012.

Figure 4 shows daily (black line) and expected (dotted line) mortality and mean temperature (grey line) trends, the shaded area represents the cold spell episode. In Bologna, Milano, Torino and Roma excess daily deaths were concomitant with the decline in temperatures and remained high through to the end of the month. Conversely, in Genova and Trieste excess mortality was observed with some delay after the cold spell event.

When we consider mortality during the entire winter season (December–March) an excess was observed in all cities that had an excess during the cold spell as well as several others (table 2). The excess ranged from +12% in Firenze and Venezia to +89% in Latina. These results suggest that during winter 2011–2012 mortality was above average and that the excess observed in February was not entirely compensated by a reduction in deaths in the following month.

**Table 2:** Observed, excess and percent variation in mortality among the very old (75 years and over) during the winter season (December 2011–March 2012) in major Italian cities.

City	Observed	Excess	% variation	P-value
Bolzano	282	45	**19**	**0.007**
Torino	2288	387	**20**	**<0.001**
Milano	3372	513	**18**	**<0.001**
Brescia	554	104	**23**	**<0.001**
Verona	734	114	**18**	**<0.001**
Venezia	976	102	**12**	**0.001**
Trieste	845	106	**14**	**<0.001**
Genova	2298	270	**13**	**<0.001**
Bologna	1297	140	**12**	**<0.001**
Firenze	1257	147	**13**	**<0.001**
Ancona	268	−8	−3	0.625
Perugia	449	139	**45**	**<0.001**
Roma	6617	819	**14**	**<0.001**
Viterbo	162	29	22	**0.023**
Rieti	110	16	17	0.127
Civitavecchia	140	24	**21**	**0.043**
Frosinone	113	0	0	1
Latina	215	101	**89**	**<0.001**
Pescara	333	66	**25**	**<0.001**
Campobasso	106	17	19	0.099
Napoli	2430	580	**31**	**<0.001**
Cagliari	354	13	4	0.49
Bari	571	−13	−2	0.586
Reggiocalabria	498	51	**11**	**0.022**
Messina	687	27	4	0.303
Palermo	1521	401	**36**	**<0.001**

Table 3 shows the excess in mortality in Roma by cause of death in the old (65+ years) and very old (75+ years). The highest excess in deaths was registered for respiratory disease in both age groups (+63 and 64% respectively). For this cause of death, most of the excess was coded as COPD (+58% and +57%, respectively for the two age groups) and to a lesser extent to acute respiratory infections (+56% and +58%, respectively for the two age groups). A statistically significant excess in cardiovascular disease was also observed (+20% in the 75+ age group). For ischemic heart disease, it is interesting to note that a greater excess was observed among the 65+ age group (+20% versus +14% in the 75+ age group). Other heart disease also registered a statistically significant increase in both old (+37%) and very old (+33%) age groups (Table 2).

**Table 3:** Monthly observed, excess and percent variation in cause-specific mortality among the old (65+ years) and very old (75+ years) during February 2012 in Roma.

	65+					75+			
	ICD-IX	observed	excess	% var	p_value	observed	excess	% var	p_value
**All natural causes**	1-799	2136	480	**29**	**<0.001**	1765	434	**33**	**<0.001**
**Cardiovascular disease**	390-459	876	142	**19**	**<0.001**	775	127	**20**	**<0.001**
Ischemic Heart Disease	410-414	310	51	**20**	**0.003**	257	32	**14**	**0.045**
*Acute myocardial infarction*	410-412	128	12	10	0.3	97	1	1	0.941
other heart disease	420-429	205	51	**37**	**<0.001**	185	50	**33**	**<0.001**
*cardiac dysrhythmias*	427	43	11	34	0.093	37	7	23	0.250
*heart failure*	428	35	5	17	0.398	32	4	14	0.480
**Respiratory disease**	460-519	220	85	**63**	**<0.001**	191	74	**64**	**<0.001**
Acute respiratory infections	(464-466),(480-487)	30	11	**56**	**0.05**	26	9	58	0.063
Chronic obstructive pulmonary disease	490-496	148	54	**58**	**<0.001**	129	47	**57**	**<0.001**
**Injuries**	800-959	66	4	6	0.623	63	8	15	0.314

Table 4 shows the number of ER visits in all ER departments of Roma among the very old (75+ year olds). Although for the total number of ER visits an increase was not observed, when we considered cause-specific results a significant excess was seen for respiratory disease (186 excess visits, +23%) and in particular for acute respiratory infections (114 excess visits, +40%). A significant excess was also found for disease of the circulatory system (+9%) and among this group, the excess was mainly for other heart disease (+20%) as observed mortality. Among the latter group it is worth mentioning that ER visits were mainly for heart failure (ICD-IX CM: 428) and cardiac dysrhythmias (ICD-IX CM: 427).

**Table 4:** Monthly observed, excess and percent variation in cause-specific ER visits among the very old (75+ years) during the month of February 2012 in Roma.

	ICD-IX	Observed	Excess	var %	p_value
**All natural cases**	1-799	7072	72	1	0.392
**Disease of the circulatory system**	390-459	1831	149	**9**	**<0.001**
Ischemic Heart Disease	410-414	226	15	7	0.318
*Acute myocardial infarction*	410-412	192	9	5	0.516
Other heart disease	420-429	888	145	**20**	**<0.001**
*cardiac dysrhythmias*	427	358	31	9	0.101
*heart failure*	428	479	105	**28**	**<0.001**
**Disease of the respiratory system**	460-519	1008	186	**23**	**<0.001**
Acute respiratory infections	464-466,480-487	396	114	**40**	**<0.001**
Chronic obstructive pulmonary disease	490-496	264	55	**26**	**<0.001**
**Injuries**	800-959	1922	75	4	0.087

## Discussion

Data from the Italian mortality surveillance system allowed us to rapidly evaluate the effect of the cold spell that occurred in February 2012 on mortality. A significant excess in mortality was observed in many Italian cities as a consequence of the cold spell and throughout the winter season of 2011–2012.

Mortality trends typically show a peak during winter attributable to the seasonality of infectious disease, in particular influenza, as well as low temperatures and socio-economic factors [19]–[20], [29]. In most European countries, hypothermia represents a limited amount of total deaths related to cold and is associated to specific subgroups of the population. The elderly, small children and socially isolated individuals are considered the most susceptible to low temperatures [14], [20], [24], [29]–[31]. The elderly are particularly at risk, due to their limited thermoregulation and concomitant chronic disease and if they are not self-sufficient. Results from our analysis seem to indicate that the greatest impact was among the very old (75 years and over).

A cause-specific analysis conducted only in Roma also suggests that most deaths were for respiratory and cardiovascular outcomes. Throughout the literature there are several explanations for this. In our study, respiratory deaths and ER visits registered the greatest proportion of the excess (+64% and +20% respectively) this could be partly due to the more rapid spreading of infections. Moreover, low temperatures may reduce natural response mechanisms of the upper respiratory tract and suppress immune responses to infections. Cold weather may also exacerbate chronic respiratory disease such as asthma and bronchitis [14], [29], [30].

Cold exposure may also trigger cardiac and cerebrovascular disease in susceptible individuals through inflammatory and coagulation responses [30]. A recent study suggested that the increase in risk of myocardial infarction at colder ambient temperatures may be a driver of cold-related increases in overall mortality during winter [31].

A limit of the study is that the baseline was constructed only with 4 years of data, thus probably making it unstable, however we had no alternative as data availability was restricted to this period. On the other hand, total mortality excess estimates might be overestimated as we did not adjust for influenza epidemics. It has been suggested that seasonal trends and peaks in cardiac deaths are influenced by influenza epidemics [21], [32]–[34]. Moreover, Von Klot et al 2012 suggests that although influenza is an important risk factor for cardiac deaths, the temperature-response functions for cardiac mortality were not greatly affected when adjusting for influenza. Influenza data was not available at the municipal level for the cities included in our study in due time to be included in our analysis. A national summary report recently published indicates that the peak in influenza was during the 5^th^ week of 2012 (30jan–5feb 2012) coinciding with the beginning of the cold spell episode [35]. Due to the partial overlap in the occurrence of the cold spell and influenza peak, there is the potential for some degree of confounding by influenza in our estimates. However, when we look at the peak incidence rate value of influenza by winter season, winter 2011–2012 registered an average value compared to the annual peaks recorded in the 2004–2012 [35], hence we can exclude influenza had a dominant role on our estimates.

Seasonal dynamics and patterns in mortality are to some extent dependent on previous season mortality trends [36]–[37]. In particular, high winter mortality will deplete the pool of elderly susceptible individuals thus reducing the effect of high temperatures on mortality in the following summer period; the strong seasonality in the mortality pattern of this subgroup is partially attributable to a mortality displacement mechanism. Moreover, the pool of susceptible populations change over time also in relation to the intensity of extreme events during each season.

The impact of the February 2012 cold spell on mortality in the Italian cities suggest that cold-related mortality is still a major problem and has several public health implications. In regions where extreme cold episodes are rare events and populations are unprepared to cope with these extreme conditions prevention plans should be implemented ensuring the collaboration and cohesive management between institutions and optimizing the often limited economic resources. Similarly to heat prevention programs, a cold prevention plan should comprise city-specific warning systems, near-real time surveillance systems and local prevention measures targeted to susceptible subgroups. It is clear that timeliness in terms of interventions and preparedness is crucial; especially for health services that have to be prepared to cope with an increase in the number of patients suffering from exposure to the cold, hypothermia and accidents, as well as respiratory infections and cardiovascular diseases. Information from surveillance systems, like those considered in our study for both mortality and ER visits, may be useful in public health decision-making, improving health service response and evaluating prevention measures. The identification of susceptible subgroups vulnerable to extreme cold is important in terms of public health planning and effective prevention [38]. Clinicians and GPs should be aware of the health risks associated to the exposure to extreme temperatures and should consider this aspect in risk prevention and management.

Climate change scenarios predict a long-term trend of warming, as well as an increase in extreme events both in terms of temperatures and precipitation across Europe and in particular for the Mediterranean Basin [39]. In the last decade great attention has been paid to the impact of heat waves on health, probably due to the more frequent recurrence of these events throughout Europe. However, considering the impact of this recent cold spell on mortality in Italy, it seems important to invest in the definition of prevention plans, also for other extreme events like cold spells, even in a time of economic crisis and scarcity of economic funds especially for the health sector.
